# Supplementary material for: Genetic landscape of populations along the Silk Road: admixture and migration patterns
Source: BMC Genet. 2014 Dec 5;15:131. doi: 10.1186/s12863-014-0131-6 (PMC4267745; doi:10.1186/s12863-014-0131-6)

Supplementary figures for:

Genetic landscape of populations along the Silk Road: admixture and migration patterns

Massimo Mezzavilla[1,2] , Diego Vozzi[1], Nicola Pirastu[2], Giorgia Girotto[2], Pio d'Adamo[2], Paolo Gasparini[1,2] ,Vincenza Colonna[3]

[1] Institute for Maternal and Child Health - IRCCS "Burlo Garofolo" , Trieste, Italy

[2] Department of Medical Sciences, University of Trieste, Italy

[3] Institute of Genetics and Biophysics 'A. Buzzati-Traverso', National Research Council (CNR), Naples, Italy

Figure S1. Principal component analysis of populations from the Silk Road newly genotyped in this study (in color) with populations in the Human Genome Diversity Project panel (in grey). Silk Road populations, and especially Uzbekistan and Kazakhstan, span over a large region of the plot, suggesting great genetic diversity among individuals. The first axis explain the 5.6% of the variance, the second axis 4.3 % of the variance

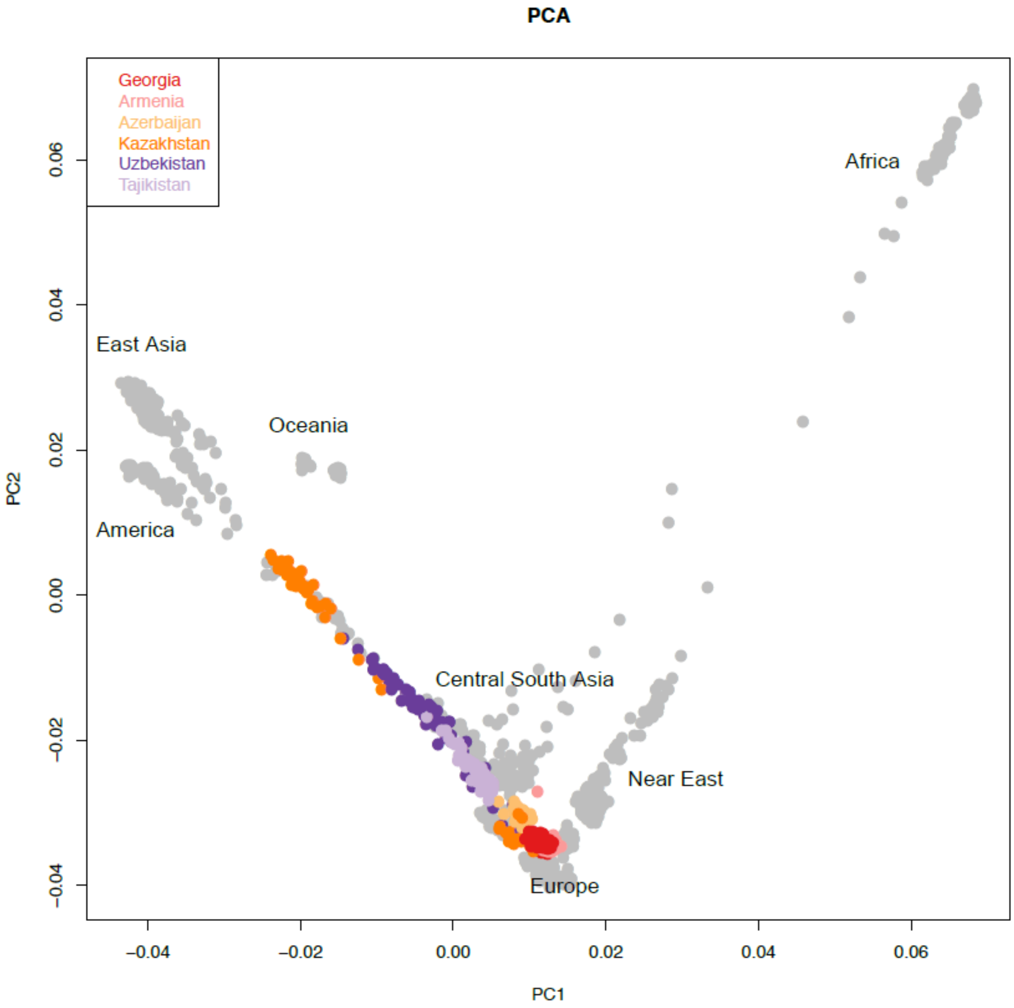

Figure S2 Admixture plots for the hypotheses of 8, 9 and 10 clusters (K) . The most likely hypothesis (K=10) shows little or no contribution from Africa, America and Oceania to populations newly genotyped in this study.

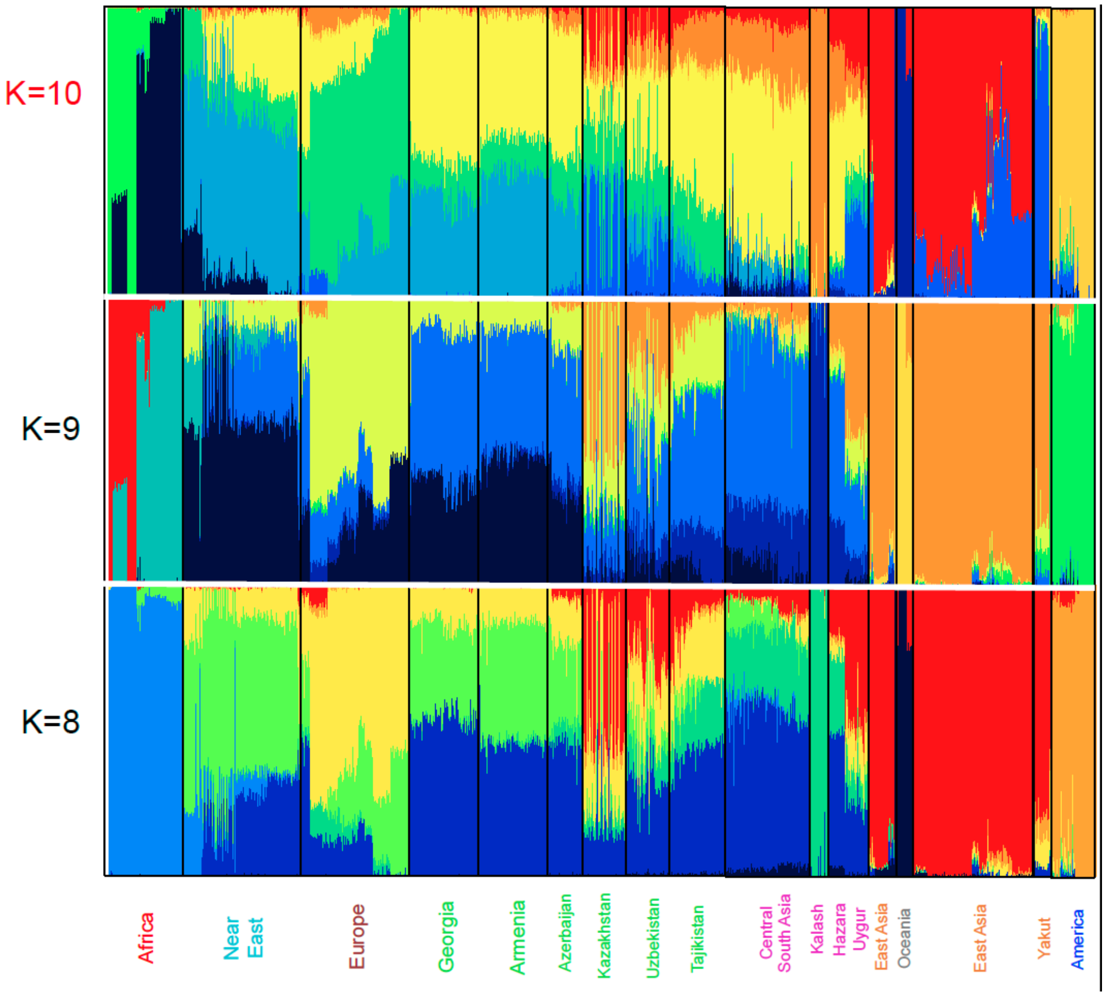

Figure S3. Principal Component Analysis of a subset of worldwide populations. Representation of components 3 to 6

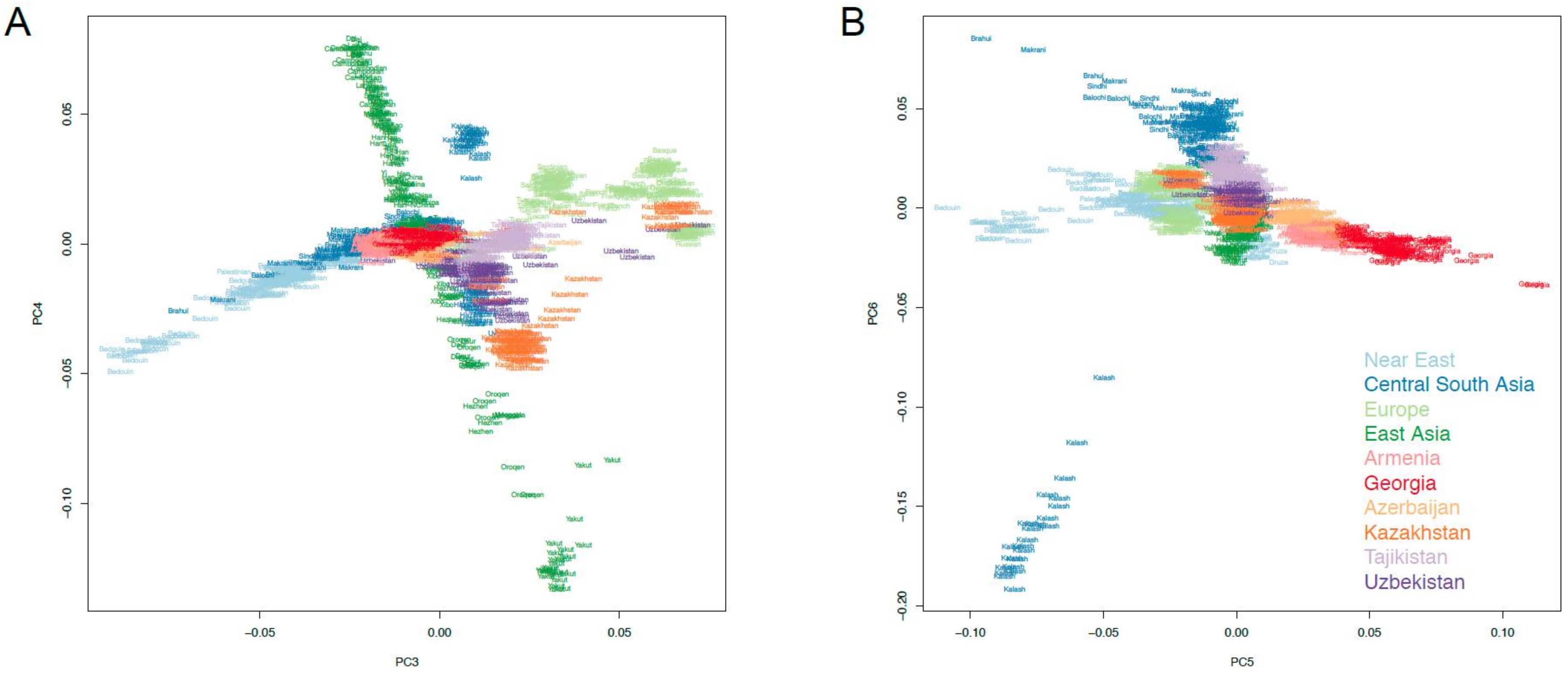

Figure S4. Admixture plots for the hypotheses of 4,6 and 8 clusters (K) in a subset of populations.

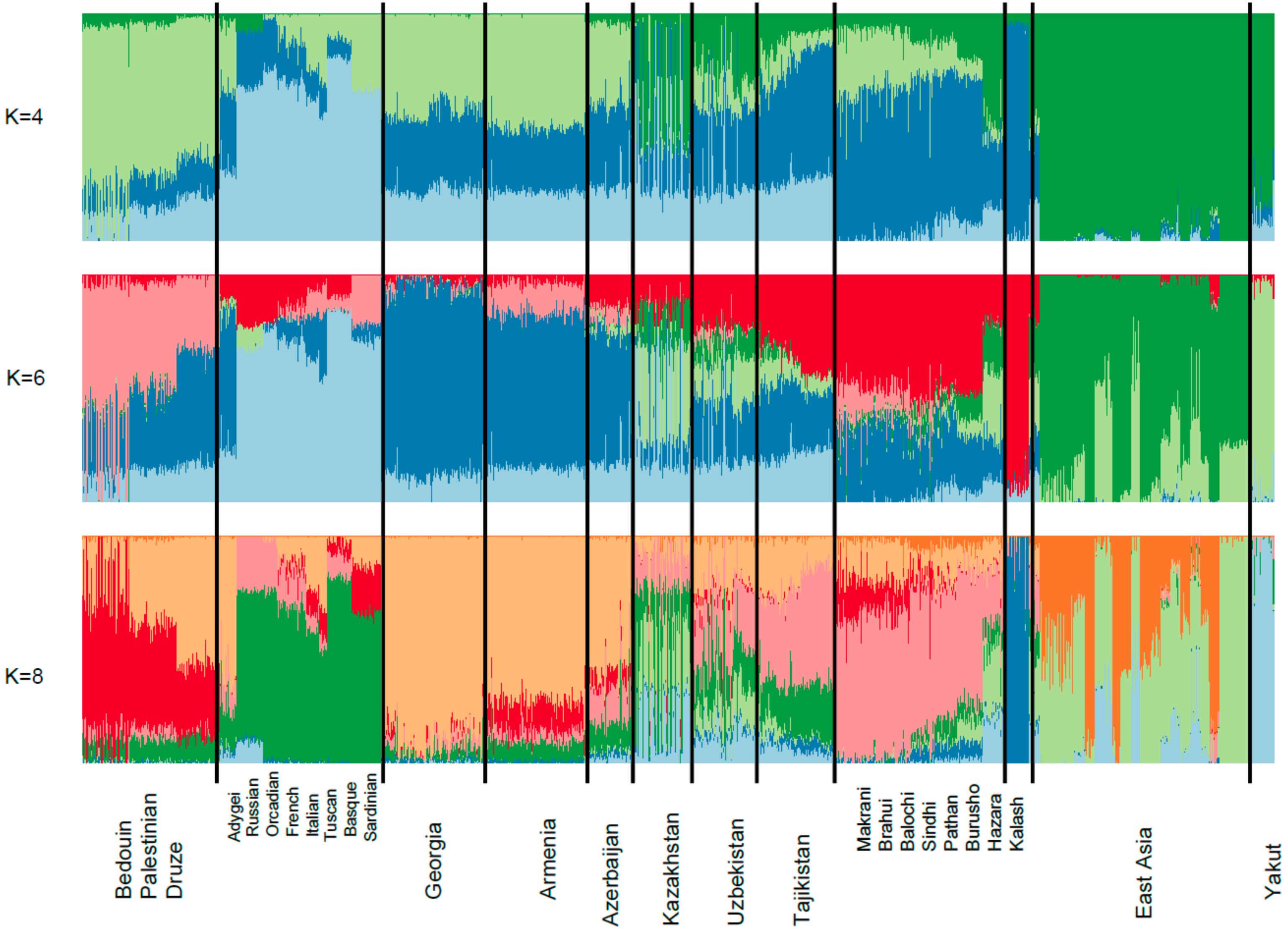

Figure S5. Inbreeding coefficients. Averages of individuals inbreeding coefficients (y-bars represent standard deviation) per population or group of populations.

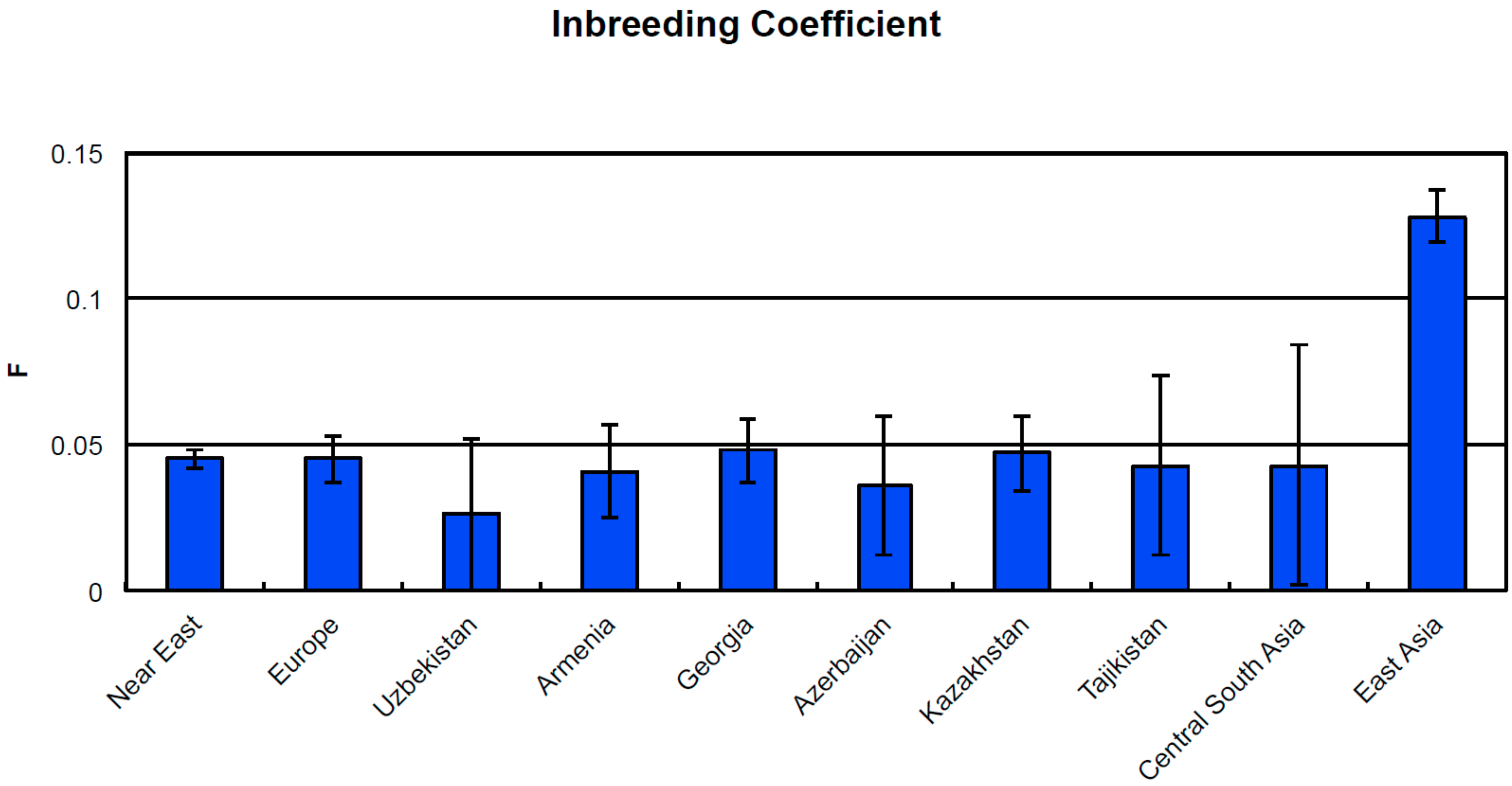

Figure S6. Cumulative distribution of runs of homozygosity. The x-axis indicate the total homozygosity in the genome in Megabases (Mb). We set the minimum ROH length to 2 Mb.

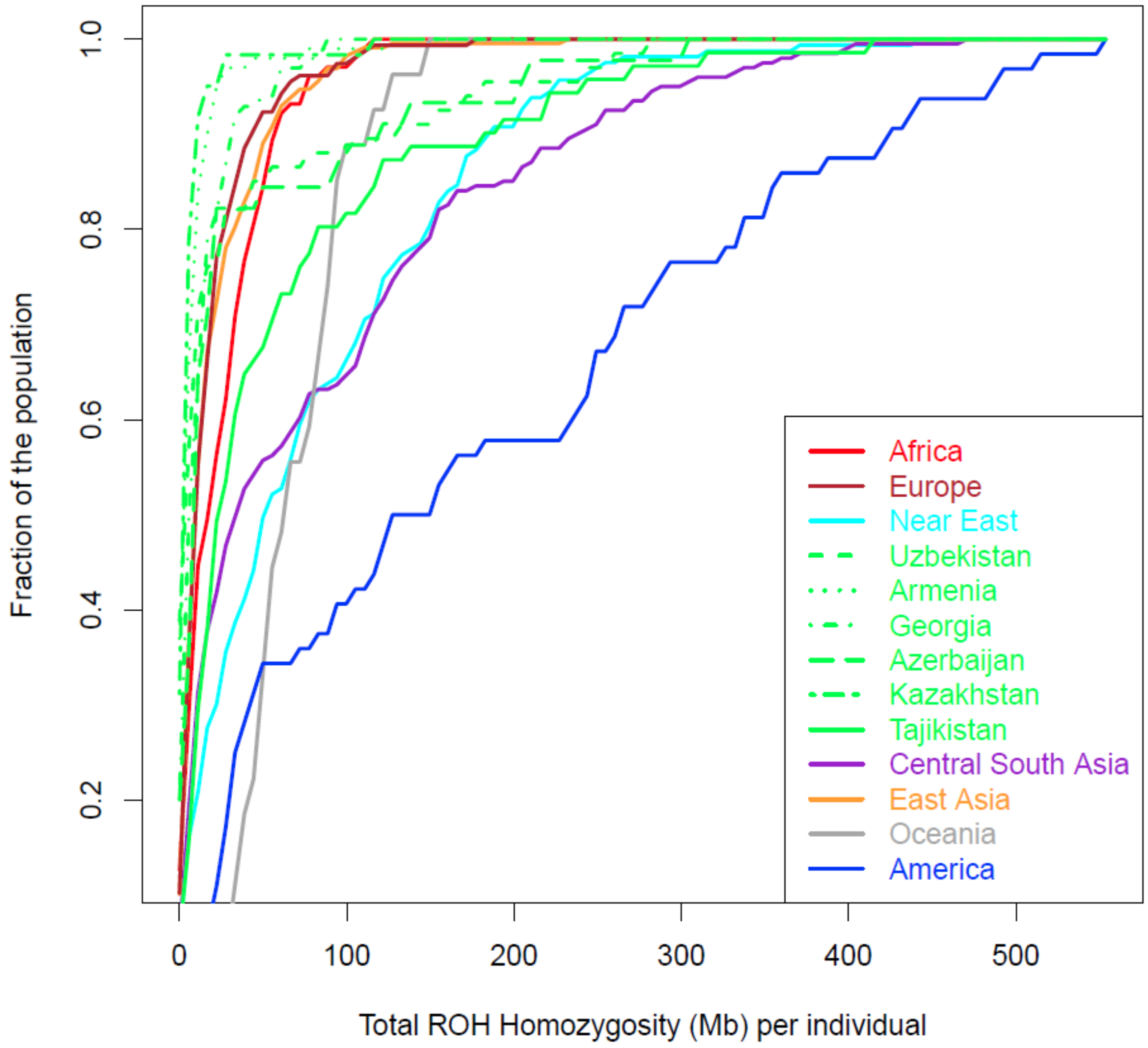

Figure S7. Effective population size through time. The y-axis represent the effective population size (Ne), the x-axis the time in the past. Generation time is 25 years

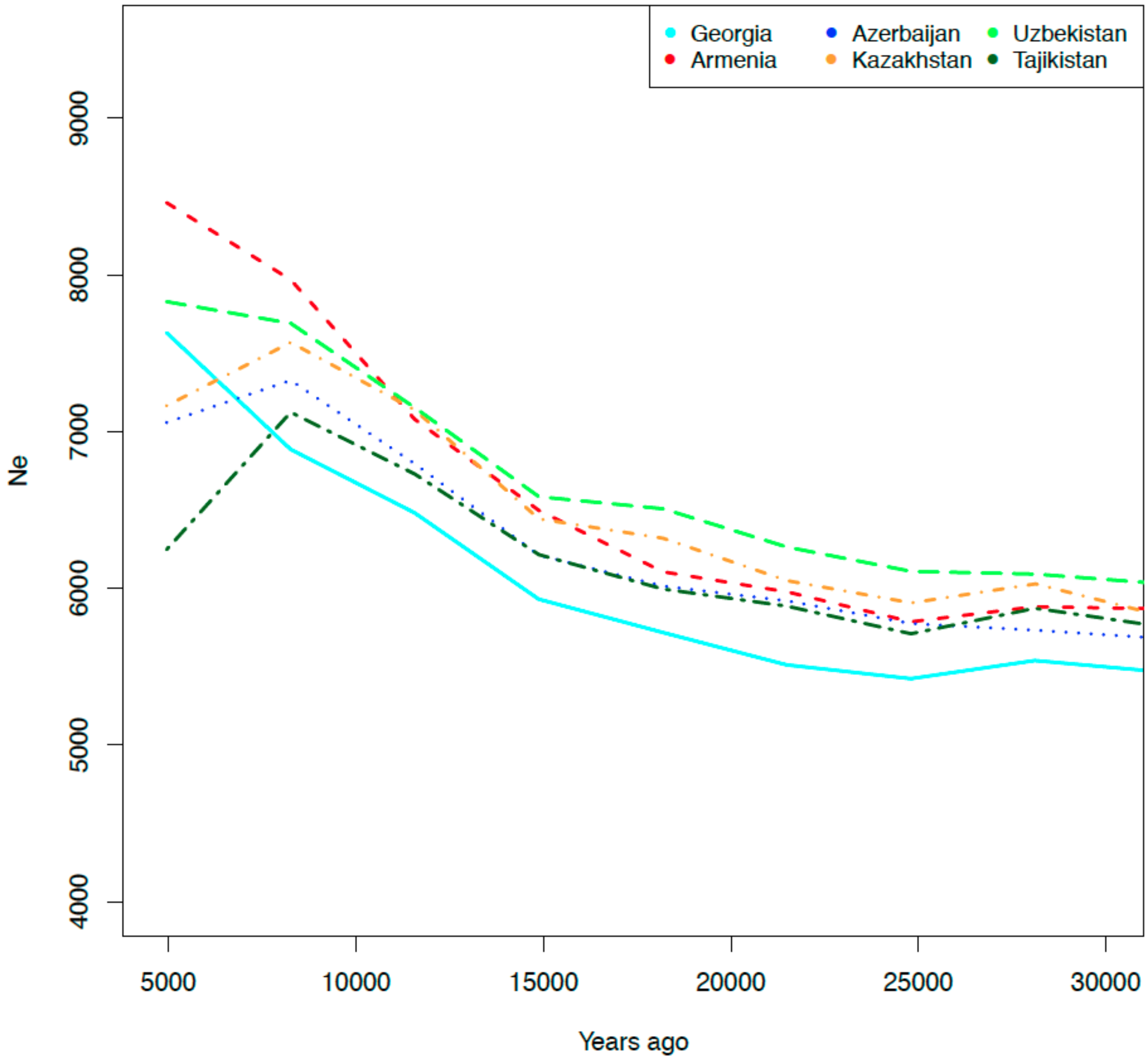

Figure S8. Long term estimate of the effective population size.

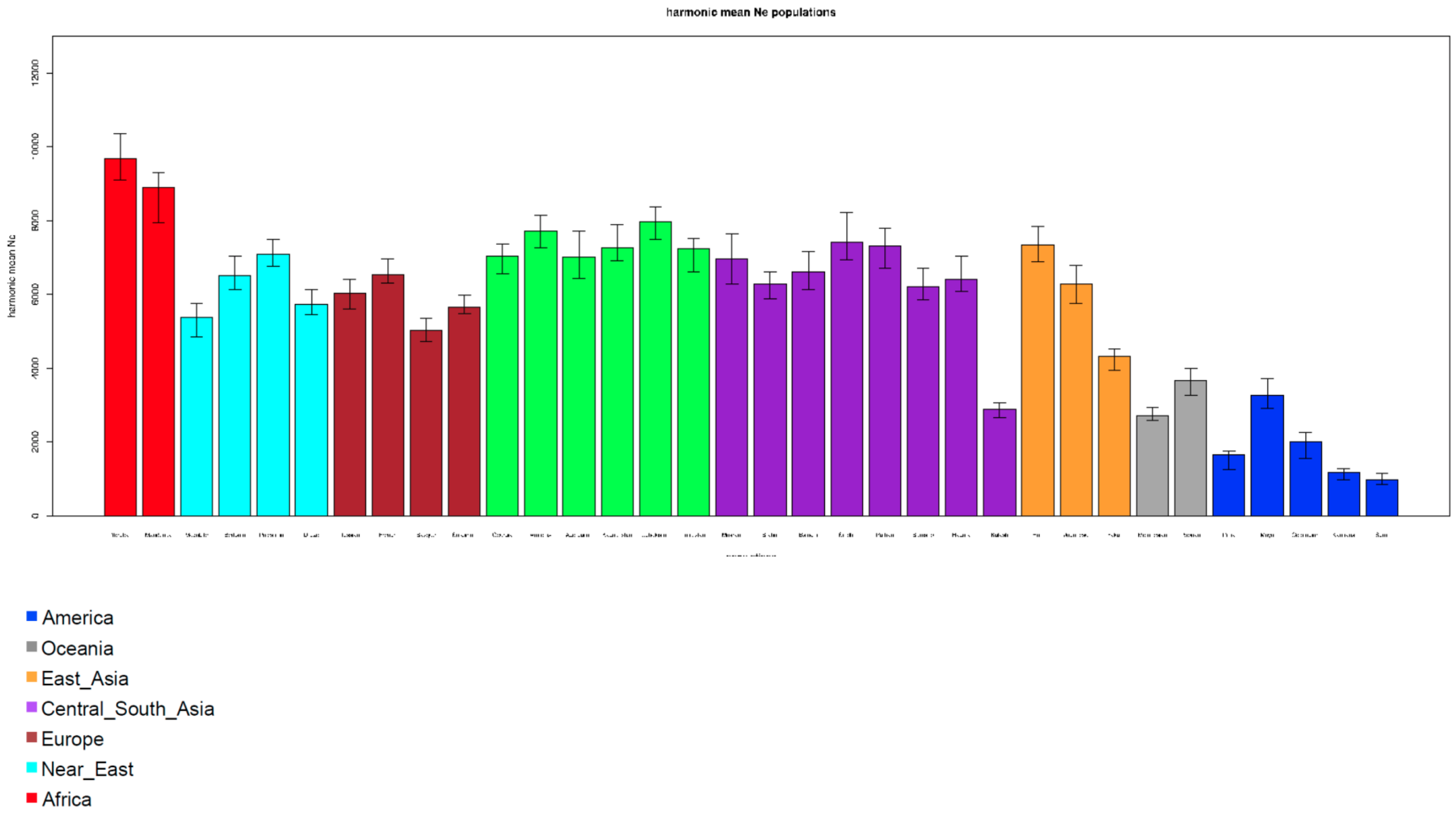

Figure S9. Proportions of admixture between pairs of populations inferred from haplotype sharing. On the y-axis the mean percentage (estimated across 22 autosomes) of genomic segment assigned to different continental groups (see colors in the legend) per each population on the x-axis.

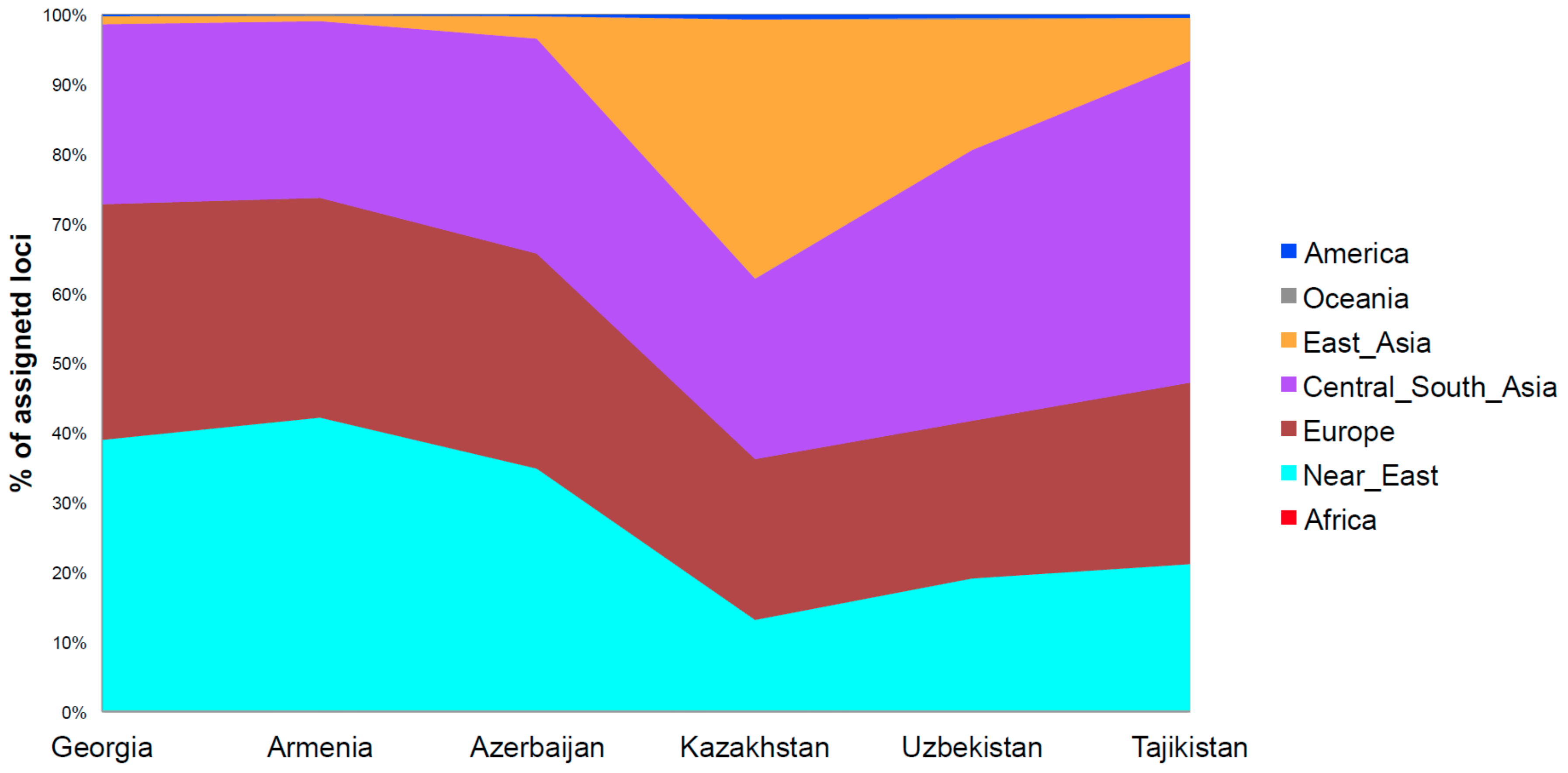

Supplement: Additional file 2: Figure S1. — Principal component analysis of populations from the Silk Road newly genotyped in this study (in color) with populations in the Human Genome Diversity Project panel (in grey). Silk Road populations, and especially Uzbekistan and Kazakhstan, span over a large region of the plot, suggesting great genetic diversity among individuals. The first axis explain the 5.6% of the variance, the second axis 4.3% of the variance. Figure S2. Admixture plots for the hypotheses of 8, 9 and 10 clusters (K). The most likely hypothesis (K=10 ) shows little or no contribution from Africa, America and Oceania to populations newly genotyped in this study. Figure S3. Principal Component Analysis of a subset of worldwide populations. Representation of components 3 to 6. Figure S4. Admixture plots for the hypotheses of 4,6 and 8 clusters (K) in a subset of populations. Figure S5. Inbreeding coefficients. Averages of individuals inbreeding coefficients (y-bars represent standard deviation) per population or group of populations. Figure S6. Cumulative distribution of runs of homozygosity. The x-axis indicate the total homozygosity in the genome in Megabases (Mb). We set the minimum ROH length to 2 Mb. Figure S7. Effective population size through time. The y-axis represent the effective population size (Ne), the x-axis the time in the past. Generation time is 25 years. Figure S8. Long term estimate of the effective population size. Figure S9. Proportions of admixture between pairs of populations inferred from haplotype sharing. On the y-axis the mean percentage (estimated across 22 autosomes) of genomic segment assigned to different continental groups (see colors in the legend) per each population on the x-axis. [file 12863_2014_131_MOESM2_ESM.pdf]
